# Development and validation of a nomogram for predicting low bone mineral density in male patients with ankylosing spondylitis

**DOI:** 10.3389/fmed.2025.1549653

**Published:** 2025-05-09

**Authors:** Xiaotong Yang, Qin Cheng, Yifan Li, Hao Tang, Xin Chen, Lijun Ma, Jing Gao, Wei Ji

**Affiliations:** ^1^Nanjing University of Chinese Medicine, Nanjing, China; ^2^Nanjing Jiangning Hospital of Chinese Medicine, Nanjing, China; ^3^Department of Rheumatology, Liyang Hospital of Chinese Medicine, Liyang, China; ^4^Department of Rheumatology, Jiangsu Province Hospital of Chinese Medicine, Nanjing, China

**Keywords:** ankylosing spondylitis, dynamic nomogram, early prevention, low bone mineral density, prediction model

## Abstract

**Objective:**

This retrospective cohort study aimed to develop and validate clinical nomogram models for predicting site-specific low bone mineral density (BMD) risk in male patients with ankylosing spondylitis (AS).

**Methods:**

This study enrolled male AS patients treated at the Rheumatology Department of Jiangsu Provincial Hospital of Traditional Chinese Medicine between January 2017 and September 2024. A total of 322 eligible patients were randomly allocated to training and validation cohorts at a 7:3 ratio. Potential predictors of low BMD at the lumbar spine (LS) and left hip (LH) were initially screened through univariate logistic regression (*p* < 0.05), followed by stepwise bidirectional multivariate logistic regression (entry criteria *p* < 0.05) to identify independent predictors for each anatomical site. Based on the regression coefficients, we developed visualized nomogram prediction models for LS and LH low BMD, accompanied by an interactive online prediction tool. The models were comprehensively evaluated for discrimination, calibration, and clinical utility. After identifying the primary predictive factors, exploratory subgroup analyses were conducted to assess effect heterogeneity of key variables (BMI and serum uric acid).

**Results:**

This study included 322 male AS patients randomly allocated to training (*n* = 225) and validation (*n* = 97) cohorts with balanced baseline characteristics (all *p* > 0.05). Multivariate logistic regression identified age at onset (LS OR = 0.96, 95%CI:0.93–0.99; LH OR = 0.97, 95%CI: 0.95–0.99), BMI (LS OR = 0.90, 95%CI: 0.81–0.99; LH OR = 0.81, 95%CI: 0.72–0.91), serum uric acid (LS/LH OR = 0.99, 95%CI: 0.99–0.99), and hip involvement (LS OR = 3.22, 95%CI: 1.71–6.05; LH OR = 8.03, 95%CI: 4.01–16.09) as common independent predictors for low BMD at both sites, while serum calcium (OR = 12.19, 95%CI: 1.44–103.25) was specific to LS. The developed nomograms, including web-based versions, demonstrated good discrimination (LS AUC: 0.77 training/0.73 validation; LH AUC: 0.82/0.85) and calibration. Decision curve analysis revealed significant net clinical benefit across probability thresholds (LS: 0.17–0.86 training/0.20–0.82 validation; LH: 0.15–0.92/0.27–0.91). The protective effect of BMI exhibited site-specific patterns: LS (low-TC: OR = 0.86; high-TC: OR = 0.77), LH (low-TC: OR = 0.77; mid-TC: OR = 0.74), with the most pronounced effect observed in the LS low-TG subgroup (OR = 0.79). SUA demonstrated consistent protective effects (LS/LH: OR = 0.95–0.99, all *p* < 0.05), potentially independent of disease stage. Interaction analyses revealed that neither lipid levels nor disease stage significantly modified the effects of BMI and SUA (all interaction *p* > 0.4).

**Conclusion:**

This study developed clinical prediction models with excellent discriminative ability and substantial clinical utility for male patients with AS. These models offer rheumatologists an efficient tool to rapidly assess individual risks of low BMD, facilitating early diagnostic decision-making and enabling personalized interventions tailored to anatomical site-specific osteoporosis risks.

## 1 Introduction

Ankylosing spondylitis (AS) represents a chronic autoimmune inflammatory disease that primarily impacts the axial skeleton and sacroiliac joints, resulting in symptoms such as inflammatory back pain, stiffness, and limited mobility (1). The pathological features of AS exhibit notable gender disparities, with a higher prevalence in males, where the male-to-female proportions fluctuate from 2:1 to 9:1 (2). Furthermore, studies have revealed that male individuals with AS exhibit higher rates of low bone mineral density (BMD) at early disease onset relative to their female counterparts (3). Low BMD is a common complication associated with AS (4), and its prevalence in male patients has been reported to range from 41.9 to 68% (5, 6). The existence of low BMD increases the likelihood of fractures, vertebral deformities, and spinal cord injuries in these patients, which can severely affect their posture and overall physical function, thus leading to a marked decline in both physical health and quality of life (7). However, the early detection of bone loss proves challenging due to its subtle progression and individual variability. Additionally, the high cost and invasiveness of dual-energy X-ray absorptiometry (DXA) have hindered its widespread use, particularly in developing regions (8). Therefore, developing a simple, effective tool for early screening of low BMD in male AS patients remains a pressing priority.

The nomogram prediction model is a statistical tool that visually represents mathematical models, which are designed to analyze multiple predictive variables for forecasting specific clinical outcomes. Displaying prediction probabilities in a graphical format offers an intuitive means of quantifying and illustrating disease risks, thereby supporting clinicians’ early diagnosis and treatment (9). Currently, nomogram models are employed to predict the diagnosis and prognosis of a variety of conditions, including colorectal cancer, heart failure, and immunoglobulin A (IgA) nephropathy. Nonetheless, there remains a gap in nomogram prediction models tailored specifically for male AS patients with low BMD.

This study specifically focuses on male AS patients to develop and validate a nomogram for predicting concomitant low BMD in this population. Furthermore, an online dynamic nomogram tool has been created to allow rheumatologists to perform efficient and convenient screening of male AS patients, thereby offering scientific and reliable evidence for early diagnosis, disease evaluation, and subsequent treatment planning for these individuals.

## 2 Materials and methods

### 2.1 Subject selection

Male individuals with AS who received treatment at Jiangsu Province Hospital of Chinese Medicine between January 2017 and September 2024 were chosen as the study participants. The inclusion criteria were as follows: 1. Meeting the modified New York criteria for AS (10); 2. Age ≥ 18 years; 3. Clear consciousness and reading ability, with the capacity to communicate independently with researchers or through relatives without barriers; 4. Voluntary participation after being informed of the study’s purpose. The exclusion criteria were: (1) Cases with >20% missing clinical data; (2) Concurrent diagnosis of other rheumatic autoimmune diseases or metabolic bone disorders (e.g., primary hyperparathyroidism); (3) Presence of severe systemic comorbidities (including but not limited to hepatic insufficiency or chronic kidney disease stage ≥3); (4) Recent exposure (within 3 months preceding enrollment) to medications with known skeletal effects (including systemic glucocorticoids at any dose, chronic heparin therapy, or enzyme-inducing antiepileptic drugs); (5) Documented history of excessive alcohol consumption (daily alcohol intake >40 g); (6) Prior total hip arthroplasty.

### 2.2 Clinical indicator information

The clinical indicators considered in this investigation were as follows: (1) basic clinicodemographic information: chronological age, body mass index (BMI), age at onset, course of disease, smoking history (cumulative cigarette consumption >100 cigarettes), alcohol history (daily ethanol intake >20 g), and history of long-term glucocorticoid use (prednisone-equivalent dose ≥5 mg/day for ≥3 consecutive months); (2) blood indicators: hemoglobin (Hb), serum uric acid (SUA), serum calcium (Ca), serum phosphorus (P), total cholesterol (TC), triglycerides (TG), comprehensive immunology panel (including immunoglobulin and complement levels), C-reactive protein (CRP), erythrocyte sedimentation rate (ESR), 25-hydroxyvitamin D [25 (OH) D], and HLA-B27 status; (3) radiological indicators: sacroiliitis grading, hip involvement, and BMD of the lumbar spine (LS) and left hip (LH).

Chronological age groups were primarily based on WHO standards but modified by data availability: young adults (18–44 years), middle-aged adults (45–64 years), and older adults (≥65 years).

Lipid parameters were categorized based on tertile distributions, with TC classified as low (<3.70 mmol/L), intermediate (3.70–4.52 mmol/L), or high (>4.52 mmol/L), and TG classified as low (<0.89 mmol/L), intermediate (0.89–1.40 mmol/L), or high (>1.40 mmol/L).

In accordance with the ASAS-2023 consensus criteria for early axial spondyloarthritis (11), early-stage AS cases in this study were strictly defined by meeting all of the following criteria: (1) fulfillment of the modified New York diagnostic criteria for AS; (2) duration of axial symptoms (including inflammatory back pain, buttock pain, or morning stiffness) ≤ 2 years; and confirmation by a board-certified rheumatologist that the symptoms were attributable to AS.

The grading of sacroiliitis was determined by radiologists using sacroiliac joint CT scans in accordance with the modified New York criteria (1984). This grading system comprises five levels, ranging from grade 0 (normal) to grade 4 (most severe) (10).

The definition of “hip involvement” was derived from previously published studies: (1) “Clinical hip involvement” was assessed by rheumatologists based on clinical symptoms, including hip pain, limited mobility, or medical records indicating either “current or previous hip arthritis”; (2) “Radiological hip involvement” was evaluated by rheumatologists using the BASRI-hip scoring system, with reference to recent (1 year) hip magnetic resonance imaging (12).

This study underwent review and was sanctioned by the Research Ethics Committee of Jiangsu Province Hospital of Chinese Medicine (Ethics number: 2023NL-135-02) and was executed per the Declaration of Helsinki.

### 2.3 Grouping method

The BMD (g/cm^2^) of the LS (L1-L4) and LH (encompassing the femoral neck, trochanter, and internal region) were assessed using DXA (Discovery W, Hologic). Utilizing the BMD measurements, patients were split into two cohorts: normal BMD and low BMD. According to the World Health Organization diagnostic criteria, patients exhibiting a T-score of less than −1 at any site were categorized as having low BMD (13).

### 2.4 Statistical analysis

Statistical analyses for this investigation were performed using Zstats software^1^ and R version 4.4.0. The normality of the data was evaluated through the Kolmogorov–Smirnov test. Normally distributed continuous variables were denoted as mean ± standard deviation, while non-normally distributed continuous variables were reported as median (interquartile range, 25th-75th percentiles). Categorical variables were summarized using frequencies and percentages. Between-group differences were evaluated utilizing *t*-tests, Mann–Whitney tests, or chi-square tests, as appropriate. Missing data were addressed using the multiple imputation method in SPSS (version 25.0).

Independent predictors of low BMD in male AS patients were identified through univariate and multivariate logistic regression analyses. Variables demonstrating statistical significance (*p* < 0.05) in the univariate logistic regression analysis were included in the multivariate model, with further adjustments made for clinically relevant confounders such as chronological age, smoking status, and alcohol history. Effect sizes were reported as odds ratios (OR) with 95% confidence intervals (CI), and variable selection was guided by both clinical relevance and statistical criteria. To assess the robustness of core predictors, we compared effect estimates between the primary and confounder-adjusted models. Stability was quantified using relative OR change rates:


$$\mathrm{Relative Change}\left(\%\right)=\left|\frac{ORadjusted-ORprimary}{ORprimary}\right|\times 100$$

Predictors with change rates <20% were considered stable, while directional consistency was required for those exceeding this threshold. Multicollinearity was assessed using generalized variance inflation factors (GVIF), with GVIF^[1/(2 × Df)] values <2 considered acceptable. Furthermore, subgroup analyses were performed to investigate potential effect modification by disease stage and metabolic factors on key predictor variables. Based on the final set of independent predictors, a multi-site (LS and LH) nomogram prediction model was developed using R software. A dynamic visualization tool was subsequently created with the “shinyPredict” package and deployed on the shinyapps online platform for enhanced accessibility. Model validation encompassed discrimination assessment through receiver operating characteristic (ROC) curve analysis with area under the curve (AUC) calculation, calibration assessment via the Hosmer-Lemeshow test and calibration curves, and clinical utility evaluation using decision curve analysis (DCA) and clinical impact curves (CIC) to determine net benefit. This investigation implemented a statistical significance level of *p* < 0.05.

## 3 Results

### 3.1 Patient characteristics

We enrolled 322 male AS patients, randomly allocated into a training cohort (*n* = 225) and a validation cohort (*n* = 97) at a 7:3 ratio. Tables 1, 2 present the comparative analysis of baseline characteristics and disease-related parameters between the two cohorts, demonstrating well-balanced distributions in demographic features, basic clinical characteristics, and disease profiles (all *p* > 0.05). No significant differences were observed in chronological age distribution (*p* = 0.308), with comparable median ages at onset (30 vs. 29 years, *p* = 0.471) and median course of disease (both 9 years). BMI (25.03 ± 3.53 kg/m^2^ vs. 24.65 ± 3.11 kg/m^2^, *p* = 0.332), smoking history (44.44% vs. 43.30%, *p* = 0.849), and alcohol history (28.00% vs. 27.84%, *p* = 0.976) also showed no statistically significant differences. Regarding low BMD prevalence, the overall rates in the LS and LH were 55.9 and 56.83%, respectively. Intergroup comparisons revealed similar proportions between the training and validation cohorts for both LS (55.56% vs. 56.70%, *p* = 0.849) and LH (54.22% vs. 62.89%, *p* = 0.15). Furthermore, no significant disparities were detected in laboratory parameters—including ESR, CRP, SUA, and 25 (OH) D levels—or radiographic features such as sacroiliitis grading and hip involvement (all *p* > 0.05). This comprehensive baseline equilibrium provides robust data support for the subsequent development and validation of the predictive model.

**Table 1:** Comparison of demographic and clinical characteristics between training and validation cohorts.

Variables	Total (*n* = 322)	train (*n* = 225)	test (*n* = 97)	*p*-value
Chronological age groups, years, *n* (%)				0.308
18–44	197 (61.18)	142 (63.11)	55 (56.70)	
45–64	99 (30.75)	68 (30.22)	31 (31.96)	
≥65	26 (8.07)	15 (6.67)	11 (11.34)	
Age at onset, years (median, IQR)	30.00 (23.00, 38.00)	30.00 (23.00, 37.00)	29.00 (23.00, 39.00)	0.471
Course of disease, years (median, IQR)	9.00 (3.62, 14.00)	9.00 (3.50, 13.00)	9.00 (4.00, 18.00)	0.473
BMI, kg/m^2^ (mean ± SD)	24.77 ± 3.24	25.03 ± 3.53	24.65 ± 3.11	0.332
Smoking history, *n* (%)				0.849
Never	180 (55.90)	125 (55.56)	55 (56.70)	
Ever	142 (44.10)	100 (44.44)	42 (43.30)	
Alcohol history, *n* (%)				0.976
Never	232 (72.05)	162 (72.00)	70 (72.16)	
Ever	90 (27.95)	63 (28.00)	27 (27.84)	
Lumbar spine, *n* (%)				0.849
Normal BMD	142 (44.10)	100 (44.44)	42 (43.30)	
Low BMD	180 (55.90)	125 (55.56)	55 (56.70)	
Left hip, *n* (%)				0.15
Normal BMD	139 (43.17)	103 (45.78)	36 (37.11)	
Low BMD	183 (56.83)	122 (54.22)	61 (62.89)	

Data are presented as the mean ± standard deviation (SD), median (interquartile range), or number (percentage). BMI, Body mass index.

**Table 2:** Comparison of disease-related variables between training and validation cohorts.

Variables	Total (*n* = 322)	Train (*n* = 225)	Test (*n* = 97)	*p*-value
Hb, g/L (median, IQR)	140.00 (128.00, 150.00)	141.00 (129.00, 151.00)	138.00 (125.00, 150.00)	0.307
Ca, mmol/L (median, IQR)	2.37 (2.26, 2.46)	2.38 (2.27, 2.47)	2.36 (2.24, 2.44)	0.211
P, mmol/L (median, IQR)	1.06 (0.93, 1.19)	1.06 (0.92, 1.20)	1.05 (0.94, 1.18)	0.561
SUA, μmol/L (median, IQR)	357.50 (314.00, 413.70)	357.00 (316.00, 403.00)	365.00 (297.00, 424.00)	0.507
IgG, g/L (median, IQR)	12.20 (10.50, 14.70)	12.00 (10.50, 14.80)	12.50 (10.60, 14.20)	0.874
IgA, g/L (median, IQR)	2.90 (2.13, 3.98)	2.83 (2.14, 3.98)	2.91 (2.07, 3.97)	0.721
IgM, g/L (median, IQR)	0.91 (0.69, 1.27)	0.90 (0.69, 1.25)	0.91 (0.68, 1.31)	0.986
C3, g/L (median, IQR)	1.01 (0.88, 1.18)	1.01 (0.88, 1.19)	1.00 (0.89, 1.16)	0.607
C4, g/L (median, IQR)	0.24 (0.21, 0.29)	0.24 (0.20, 0.28)	0.25 (0.21, 0.29)	0.91
CRP, mg/L (median, IQR)	9.97 (4.14, 24.62)	9.55 (4.23, 22.20)	11.00 (3.31, 30.50)	0.469
ESR, mm/h	23.00 (9.00, 44.00)	22.00 (9.00, 43.00)	25.00 (10.00, 45.00)	0.557
25 (OH) D, ng/mL (median, IQR)	20.96 (16.00, 26.00)	21.00 (16.00, 26.00)	20.00 (17.00, 25.00)	0.742
TC groups, mmol/L, *n* (%)				0.408
<3.70	107 (33.23)	78 (34.67)	29 (29.90)	
3.70–4.52	109 (33.85)	71 (31.56)	38 (39.18)	
>4.52	106 (32.92)	76 (33.78)	30 (30.93)	
TC groups, mmol/L, *n* (%)				0.217
<0.89	107 (33.23)	69 (30.67)	38 (39.18)	
0.89–1.40	109 (33.85)	76 (33.78)	33 (34.02)	
>1.40	106 (32.92)	80 (35.56)	26 (26.80)	
HLA-B27, *n* (%)				0.137
Negative	34 (10.56)	20 (8.89)	14 (14.43)	
Positive	288 (89.44)	205 (91.11)	83 (85.57)	
Sacroiliitis average, *n* (%)				0.747
2	143 (44.41)	102 (45.33)	41 (42.27)	
2.5	6 (1.86)	4 (1.78)	2 (2.06)	
3	76 (23.60)	49 (21.78)	27 (27.84)	
3.5	15 (4.66)	12 (5.33)	3 (3.09)	
4	82 (25.47)	58 (25.78)	24 (24.74)	
Hip involvement, *n* (%)				0.245
No	185 (57.45)	134 (59.56)	51 (52.58)	
Yes	137 (42.55)	91 (40.44)	46 (47.42)	
Patients on GC, *n* (%)				1
No	311 (96.58)	217 (96.44)	94 (96.91)	
Yes	11 (3.42)	8 (3.56)	3 (3.09)	

Data are presented as the mean ± standard deviation (SD), median (interquartile range), or number (percentage). Hb, Hemoglobin; SUA, Serum uric acid; Ca, Serum calcium; P, Serum phosphorus; TC, Total cholesterol; TG, Triglycerides; IgG, Immunoglobulin G; IgA, Immunoglobulin A; IgM, Immunoglobulin M; C3, Complement component 3; C4, Complement component 4; CRP, C-reactive protein; ESR, Erythrocyte sedimentation rate; 25 (OH) D, 25-hydroxyvitamin D.

### 3.2 Results of univariate and multivariate analysis

The univariate logistic regression analysis demonstrated that chronological age, age at onset, BMI, serum calcium, SUA, and hip involvement were significantly associated with low BMD at the LS (*p* < 0.05). These associations persisted in the multivariate logistic regression analysis, with age at onset (OR = 0.96, 95%CI:0.93–0.99), BMI (OR = 0.97, 95%CI:0.95–0.99), serum calcium (OR = 12.19, 95%CI: 1.44–103.25), SUA (OR = 0.99, 95%CI:0.99–0.99), and hip involvement (OR = 3.22, 95%CI: 1.71–6.05) remaining independently predictive of LS low BMD (Table 3).

**Table 3:** Univariate and multivariate logistic regression analysis of LS training cohort.

Variables	Univariate regression		Multivariate regression	
	OR (95%CI)	*p*-value	OR (95%CI)	*p*-value
Chronological age groups				
18–44	1.00 (Reference)			
45–64	0.47 (0.26, 0.85)	**0.012**		
≥65	0.40 (0.13, 1.18)	0.096		
Age at onset	0.95 (0.93, 0.98)	**<0.001**	0.96 (0.93, 0.99)	0.003
Course of disease	1.03 (0.99, 1.06)	0.152		
BMI	0.86 (0.79, 0.95)	**0.002**	0.90 (0.81, 0.99)	0.035
Hb	1.01 (0.99, 1.02)	0.248		
Ca	9.05 (1.56, 52.60)	**0.014**	12.19 (1.44, 103.25)	0.022
P	0.83 (0.24, 2.93)	0.774		
SUA	0.99 (0.99, 0.99)	**0.006**	0.99 (0.99, 0.99)	0.011
IgG	0.97 (0.90, 1.04)	0.422		
IgA	0.96 (0.80, 1.16)	0.699		
IgM	1.23 (0.80, 1.91)	0.349		
C3	2.62 (0.76, 9.07)	0.129		
C4	2.06 (0.13, 31.57)	0.605		
CRP	1.00 (0.99, 1.01)	0.414		
ESR	1.00 (0.99, 1.01)	0.727		
25 (OH) D	1.01 (0.98, 1.04)	0.687		
TC groups				
<3.70	1.00 (Reference)			
3.70–4.52	1.12 (0.58, 2.16)	0.732		
>4.52	0.95 (0.50, 1.81)	0.872		
TG groups				
<0.89	1.00 (Reference)			
0.89–1.40	1.05 (0.55, 2.01)	0.882		
>1.40	1.01 (0.53, 1.90)	0.987		
HLA-B27				
Negative	1.00 (Reference)			
Positive	1.59 (0.63, 4.01)	0.323		
Sacroiliitis average				
2	1.00 (Reference)			
2.5	2.88 (0.29, 28.66)	0.366		
3	1.98 (0.97, 4.04)	0.06		
3.5	1.92 (0.54, 6.79)	0.31		
4	0.96 (0.50, 1.83)	0.905		
Hip involvement				
No	1.00 (Reference)		1.00 (Reference)	
Yes	3.66 (2.05, 6.52)	**<0.001**	3.22 (1.71, 6.05)	<0.001
Smoking history				
Never	1.00 (Reference)			
Ever	0.77 (0.45, 1.31)	0.337		
Alcohol history				
Never	1.00 (Reference)			
Ever	0.84 (0.47, 1.50)	0.55		
Patients on GC				
No	1.00 (Reference)			
Yes	0.47 (0.11, 2.00)	0.306		

OR, Odds ratio; CI, Confidence interval. Hb, Hemoglobin; SUA, Serum uric acid; Ca, Serum calcium; P, Serum phosphorus; TC, Total cholesterol; TG, Triglycerides; IgG, Immunoglobulin G; IgA, Immunoglobulin A; IgM, Immunoglobulin M; C3, Complement component 3; C4, Complement component 4; CRP, C-reactive protein; ESR, Erythrocyte sedimentation rate; 25 (OH) D, 25-hydroxyvitamin D. Bold values indicate statistical significance (*p* < 0.05) in the univariate logistic regression results.

For low BMD at the LH, univariate analysis identified course of disease, age at onset, BMI, SUA, hip involvement, and sacroiliitis grade (specifically grade 3 versus 4) as significant predictors (*p* < 0.05). Subsequent multivariate analysis confirmed age at onset (OR = 0.97, 95%CI: 0.95–0.99), BMI (OR = 0.81, 95%CI: 0.72–0.91), SUA (OR = 0.99, 95%CI: 0.99–0.99), and hip involvement (OR = 8.03, 95%CI: 4.01–16.09) as independent predictors for LH low BMD (Table 4).

**Table 4:** Univariate and multivariate logistic regression analysis of LH training cohort.

Variables	Univariate regression		Multivariate regression	
	OR (95%CI)	*p*-value	OR (95%CI)	*p*-value
Chronological age groups				
18–44	1.00 (Reference)			
45–64	0.63 (0.35, 1.13)	0.121		
≥65	1.51 (0.49, 4.63)	0.475		
Age at onset	0.97 (0.95, 0.99)	**0.014**	0.97 (0.95, 0.99)	0.035
Course of disease	1.07 (1.03, 1.11)	**<0.001**		
BMI	0.80 (0.72, 0.88)	**<0.001**	0.81 (0.72, 0.91)	<0.001
Hb	1.00 (0.98, 1.01)	0.55		
Ca	5.49 (0.98, 30.87)	0.053		
P	0.43 (0.12, 1.53)	0.191		
SUA	0.99 (0.99, 0.99)	**<0.001**	0.99 (0.99, 0.99)	0.023
IgG	1.00 (0.93, 1.07)	0.922		
IgA	1.12 (0.93, 1.35)	0.245		
IgM	1.12 (0.78, 1.61)	0.534		
C3	1.08 (0.32, 3.65)	0.897		
C4	0.30 (0.02, 4.73)	0.39		
CRP	1.00 (0.99, 1.01)	0.929		
ESR	1.00 (0.99, 1.01)	0.867		
25 (OH) D	0.98 (0.95, 1.01)	0.277		
TC groups				
<3.70	1.00 (Reference)			
3.70–4.52	0.84 (0.44, 1.63)	0.613		
>4.52	0.65 (0.34, 1.24)	0.194		
TC groups				
<0.89	1.00 (Reference)			
0.89–1.40	1.23 (0.64, 2.34)	0.537		
>1.40	1.17 (0.62, 2.21)	0.621		
HLA-B27				
Negative	1.00 (Reference)			
Positive	1.50 (0.60, 3.78)	0.388		
Sacroiliitis average				
2	1.00 (Reference)			
2.5	1.43 (0.19, 10.55)	0.727		
3	2.26 (1.12, 4.53)	**0.022**		
3.5	2.86 (0.81, 10.11)	0.103		
4	3.17 (1.61, 6.28)	**<0.001**		
Hip involvement				
No	1.00 (Reference)		1.00 (Reference)	
Yes	8.68 (4.55, 16.55)	**<0.001**	8.03 (4.01, 16.09)	<0.001
Smoking history				
Never	1.00 (Reference)			
Ever	1.06 (0.62, 1.79)	0.834		
Alcohol history				
Never	1.00 (Reference)			
Ever	1.08 (0.60, 1.93)	0.802		
Patients on GC				
No	1.00 (Reference)			
Yes	0.84 (0.20, 3.44)	0.807		

OR, Odds ratio; CI, Confidence interval. Hb, Hemoglobin; SUA, Serum uric acid; Ca, Serum calcium; P, Serum phosphorus; TC, Total cholesterol; TG, Triglycerides; IgG, Immunoglobulin G; IgA, Immunoglobulin A; IgM, Immunoglobulin M; C3, Complement component 3; C4, Complement component 4; CRP, C-reactive protein; ESR, Erythrocyte sedimentation rate; 25 (OH) D, 25-hydroxyvitamin D. Bold values indicate statistical significance (*p* < 0.05) in the univariate logistic regression results.

Prior to conducting the multivariate logistic regression, we performed collinearity diagnostics on all variables that showed significance in the univariate analysis. The results indicated no substantial multicollinearity concerns, with all variables demonstrating GVIF^[1/(2 × Df)] values below 1.5 in both the LS and LH models (Supplementary Tables S1, S2).

### 3.3 Confounder adjustment and model robustness

To enhance the clinical applicability of our findings, we adjusted for known confounding factors including smoking history, alcohol history, glucocorticoid use, and lipid profiles in our multivariate analysis. For the LS, hip involvement (OR = 4.01, 95%CI: 2.01–8.03), BMI (OR = 0.87, 95%CI: 0.78–0.97), SUA (OR = 0.99, 95%CI: 0.99–0.99), and serum calcium (OR = 10.84, 95%CI: 1.05–112.32) emerged as significant predictors. Similar patterns were observed for the LH, where hip involvement (OR = 7.72, 95%CI: 3.51–16.98), BMI (OR = 0.80, 95%CI: 0.71–0.90), and SUA (OR = 0.99, 95%CI: 0.99–0.99) maintained their predictive value. Of particular clinical interest was our finding that elevated triglyceride levels (1.40 mmol/L) were associated with a significantly increased risk of LS BMD (OR = 3.34, 95%CI: 1.26–8.84). While age at onset exhibited site-specific effects (lumbar OR = 0.96 vs. hip OR = 0.97), traditional risk factors including smoking history, alcohol history, and glucocorticoid use showed no significant associations in either model (all *p* > 0.05). Complete details of these analyses are provided in Supplementary Tables S3, S4.

To validate the robustness of core predictors, we compared effect sizes between the primary model and the confounder-adjusted model (Supplementary Table S5). Age at onset (0% OR change), BMI (lumbar spine −3.3%, left hip −1.2%), and serum uric acid (0% change) demonstrated high stability across both models. The OR for hip involvement in the lumbar spine increased by 24.5% (3.22 → 4.01) after confounder adjustment, suggesting potential underestimation in the unadjusted model. Although serum calcium showed an 11.1% OR reduction, its wide confidence interval (1.05–112.32) indicates the need for validation in larger samples. The consistent effect directions across all variables support the clinical credibility of our models.

### 3.4 Subgroup and interaction analyses

**Figure 1:**
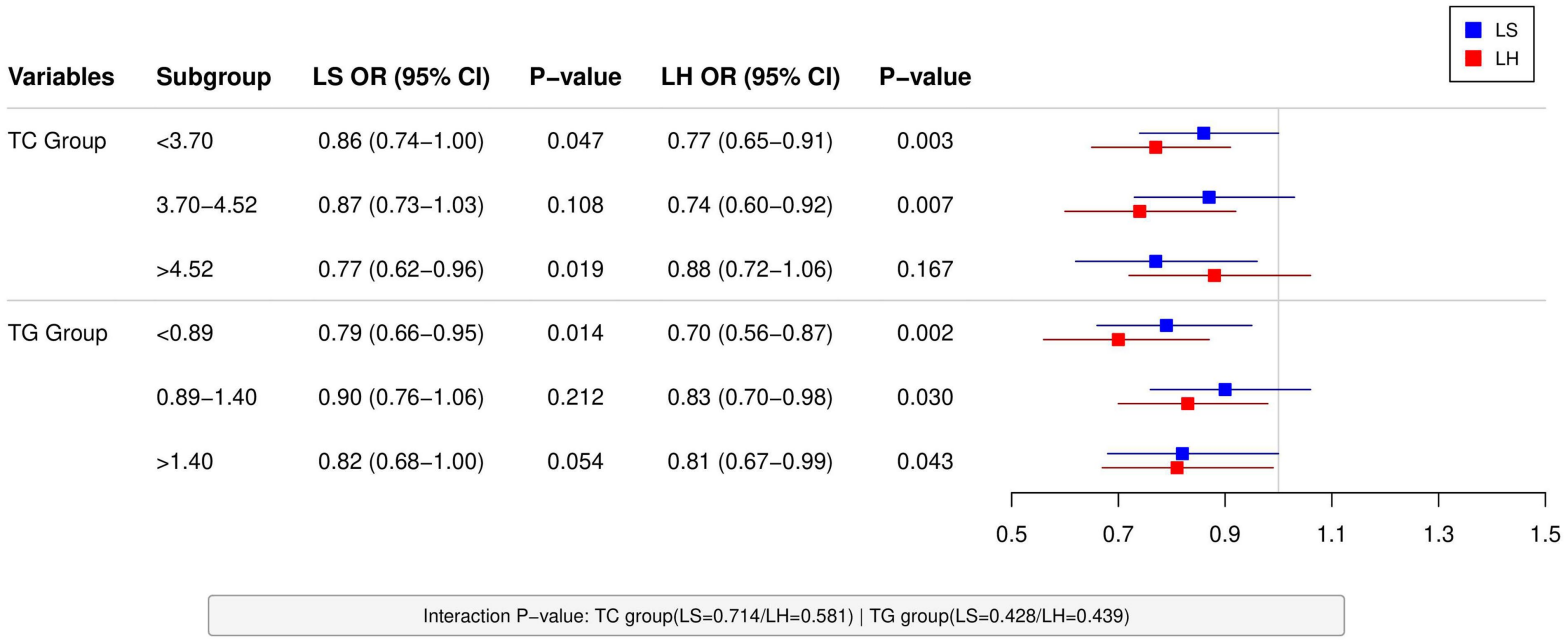
Subgroup analysis of BMI’s protective effects against low BMD in male AS patients: Integrated forest plot displays LS and LH data stratified by lipid profiles (TC/TG tertiles). Protective trends were observed in most subgroups (LS: significant in low/high-TC [OR = 0.86/0.77] and low-TG [OR = 0.79]; LH: significant in low/mid-TC [OR = 0.77/0.74] and all TG subgroups [OR = 0.70–0.83]), though nonsignificant in mid-TC for LS (*p* = 0.108). Nonsignificant interaction terms (all *p* > 0.4) suggest lipid-level-independent protective mechanisms of BMI.

BMI showed site-specific and metabolic state-dependent protective effects against low BMD in subgroup analyses. In total cholesterol (TC) stratification, BMI showed significant protective effects against LS low BMD in both low-TC (OR = 0.86, 95%CI: 0.74–1.00, *p* = 0.047) and high-TC subgroups (OR = 0.77, 95%CI: 0.62–0.96, *p* = 0.019), but not in the moderate-TC subgroup (*p* = 0.108). In contrast, its protective effect on LH low BMD was primarily observed in low- and moderate-TC subgroups (OR = 0.77 and 0.74, both *p* < 0.01). Triglyceride (TG) stratification further demonstrated that BMI’s protective effect was most pronounced for the LS in the low-TG subgroup (OR = 0.79, 95%CI: 0.66–0.95, *p* = 0.014), while maintaining significant protection for the LH across all TG subgroups (OR = 0.70–0.83, all *p* < 0.05) (Figure 1).

**Figure 2:**
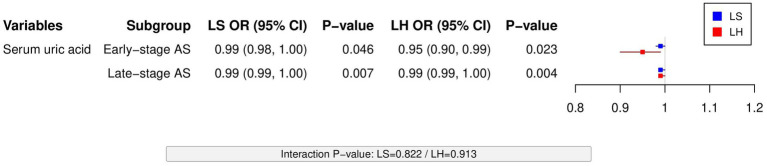
Subgroup analysis of SUA effects on low BMD: Unified forest plot presents LS and LH data across AS disease stages (early-stage: LS OR = 0.99/LH OR = 0.95; advanced-stage: LS/LH OR = 0.99). Interaction *p*-values (0.8) suggest stage-independent protection.

SUA subgroup analysis indicated its protective effect against low BMD was independent of disease stage. In early-stage AS patients, SUA showed significant associations with both LS (OR = 0.99, *p* = 0.022) and LH low BMD (OR = 0.95, *p* = 0.023). A similar trend was observed in advanced AS patients (LS: OR = 0.99, *p* = 0.007; LH: OR = 0.99, *p* = 0.004), albeit with smaller effect sizes, suggesting its clinical significance requires comprehensive evaluation with other indicators (Figure 2).

Interaction analyses demonstrated that neither lipid levels (TC interaction *p* = 0.714/0.581, TG interaction *p* = 0.428/0.439) nor disease stage (interaction *p* > 0.4) significantly modified the effects of BMI and SUA, indicating their protective roles may be independent of metabolic status and disease progression. However, it should be noted that these negative findings might be limited by sample size and statistical power, warranting future studies with larger cohorts to validate potential heterogeneity trends.

### 3.5 Development of nomogram model

**Figure 3:**
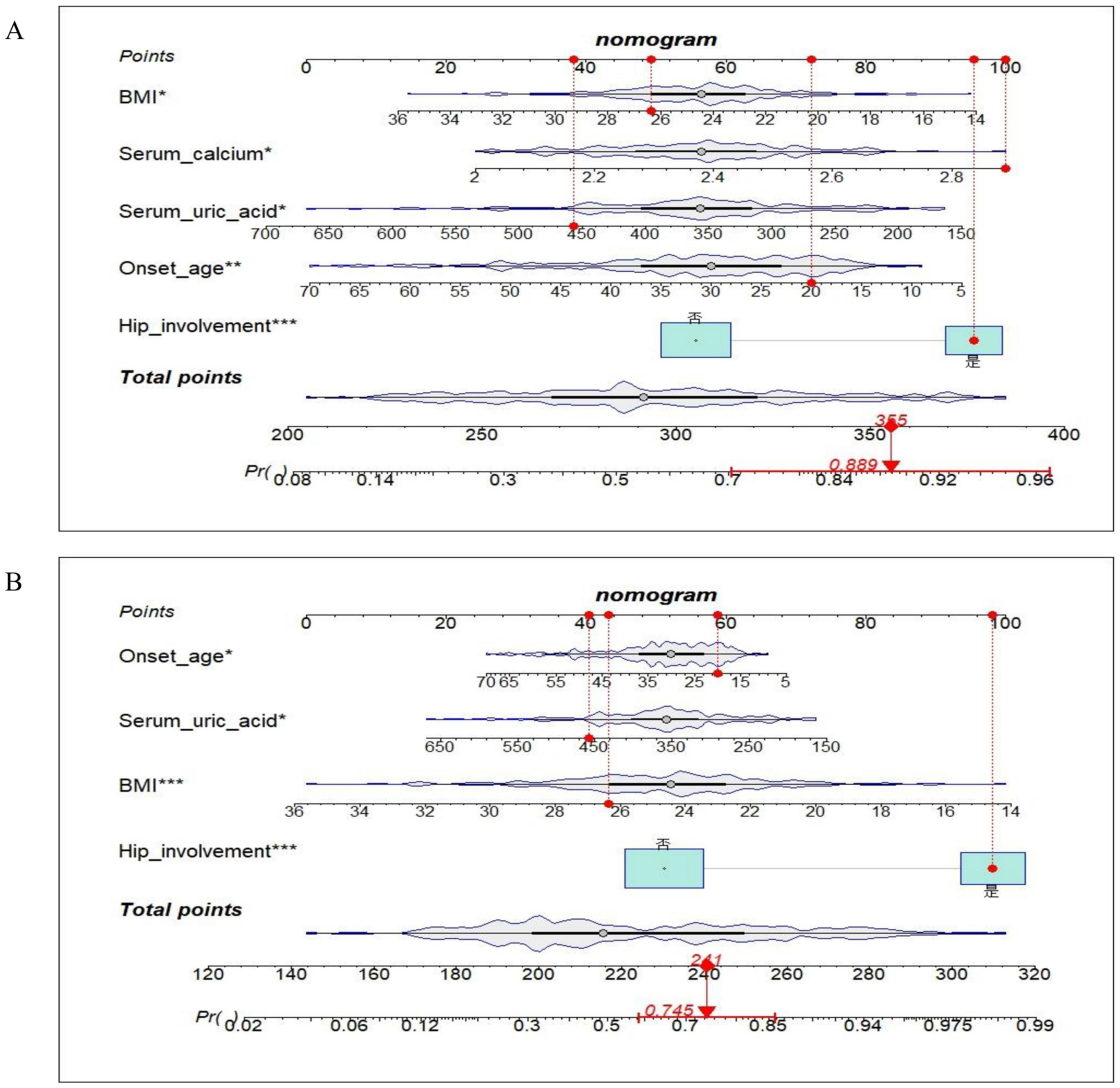
Low BMD nomograms for **(A)** LS and **(B)** LH in male AS patients, with interactive red dots for variable input (e.g., age at onset, BMI) and real-time display of total points/predicted probability (%). Example: 20-year-old male with BMI 26.35 kg/m^2^, serum calcium 2.89 mmol/L, SUA 457 μmol/L, and hip involvement (LS: 355 points → 88.9% risk; LH: 241 points → 74.5% risk).

**Figure 4:**
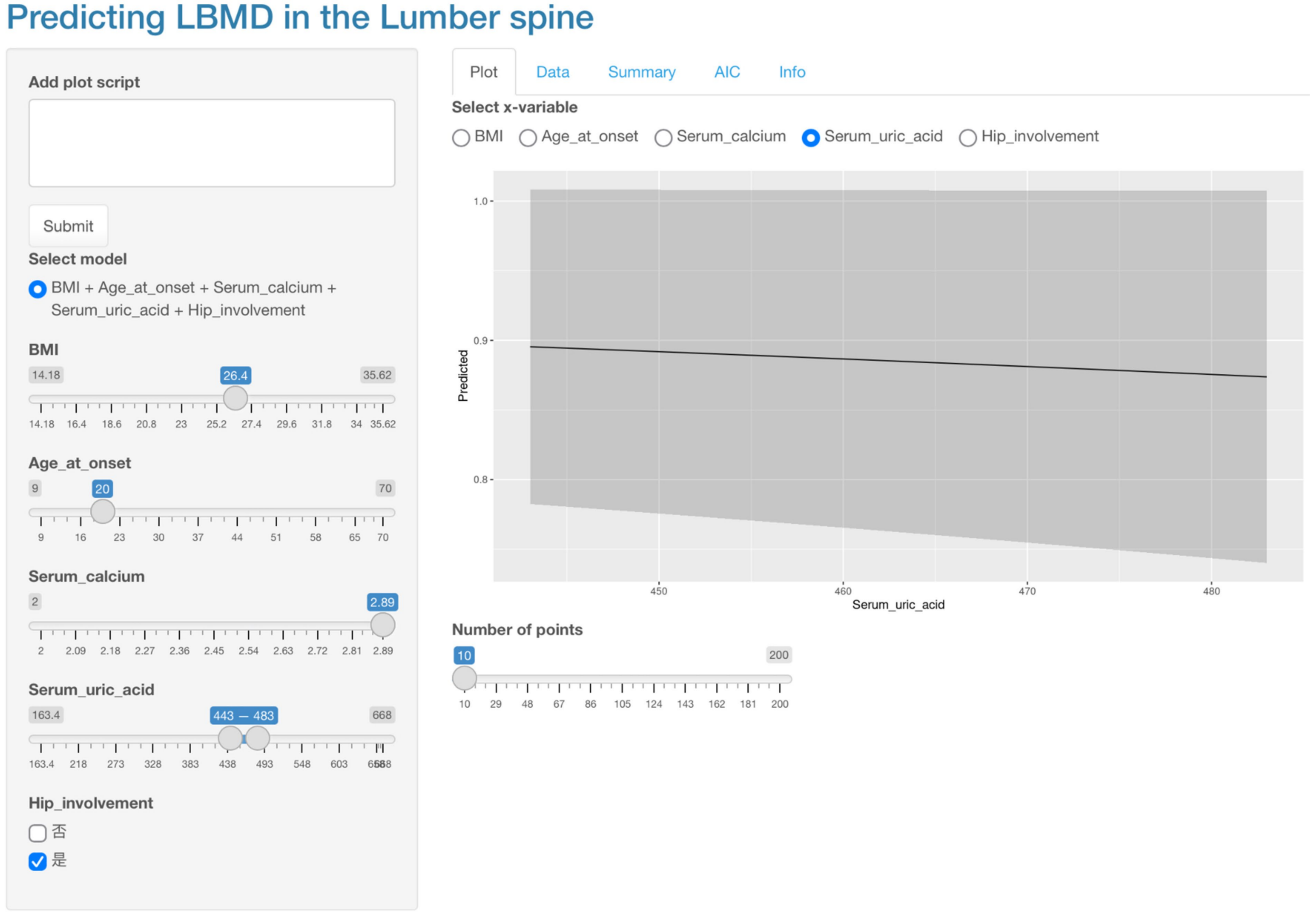
Web-based nomogram for LS low BMD prediction in male AS patients, demonstrating real-time risk probability calculation through interactive input of clinical parameters (left panel) with automated graphical output display. Operational example: Users adjust sliders for variables including age at onset, BMI, and serum biomarkers to generate instant probability estimates visualized along the scoring continuum.

Based on the outcomes of the multifactorial logistic regression analysis, clinical nomogram prediction models for the LS and LH were developed, as presented in Figure 3. Subsequently, an online dynamic nomogram prediction tool (Figure 4) was created, accessible via any device with Internet connectivity through the following links: LS: https://asresearch.shinyapps.io/shiny1/; LH: https://asresearch.shinyapps.io/shiny/.

### 3.6 Evaluation of the nomogram model

**Figure 5:**
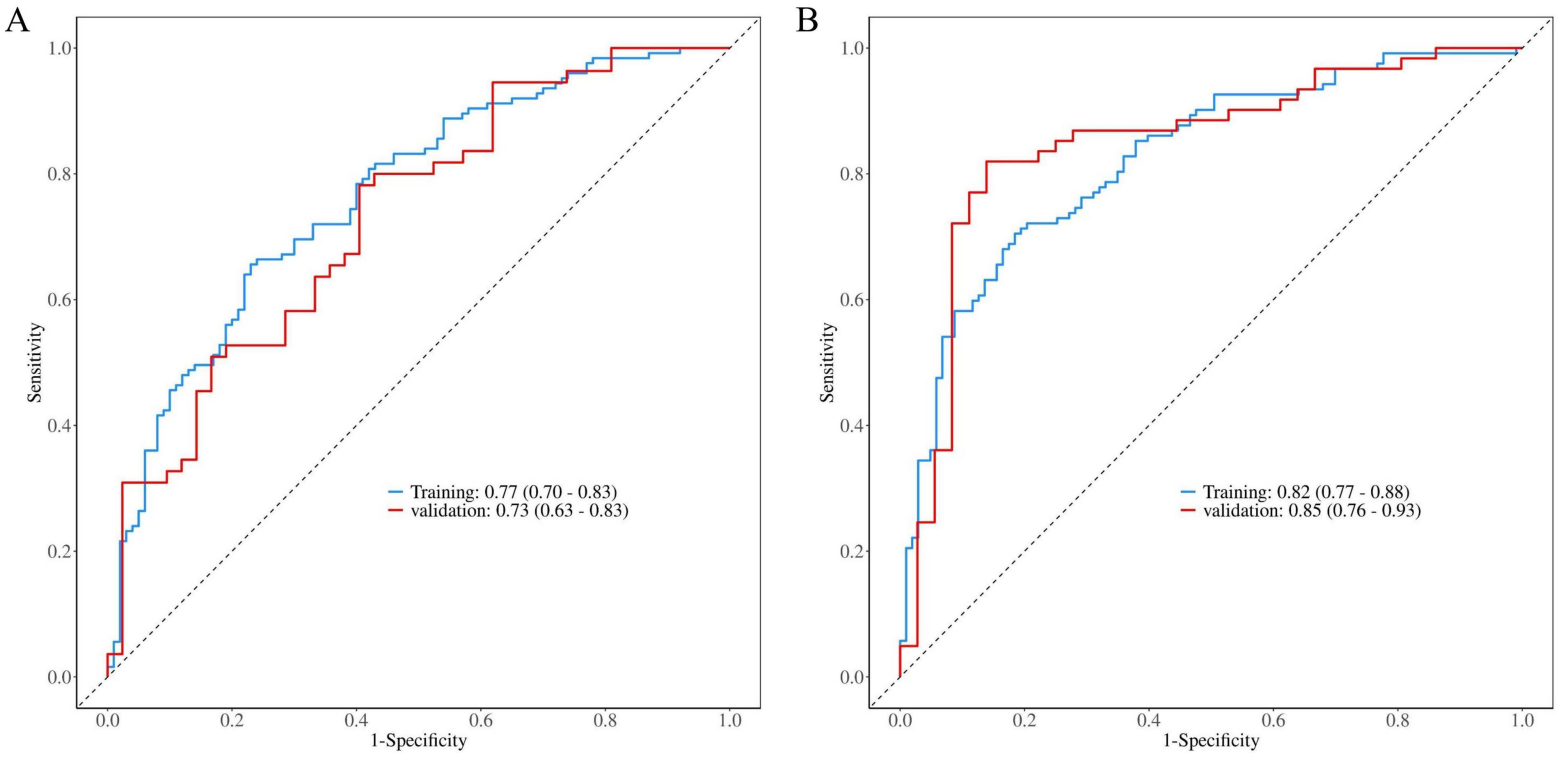
ROC analysis of low BMD prediction models for **(A)** LS and **(B)** LH, with training (blue) and validation (red) cohort performance relative to reference (dashed line, AUC = 0.5), displaying sensitivity-specificity relationships and AUC (95% CI) values.

The discriminative ability of the model was evaluated through ROC curve analysis (Figure 5). The LS low BMD prediction model demonstrated good discriminative performance in the training cohort [AUC = 0.77 (95%CI: 0.70–0.83)], with comparable results in the validation cohort [AUC = 0.73 (95%CI: 0.63–0.83)], indicating stable predictive performance of the model. The LH low BMD model exhibited even better discriminative ability, with AUC values of 0.82 (95%CI: 0.77–0.88) and 0.85 (95%CI: 0.76–0.93) in the training and validation cohorts respectively, suggesting superior predictive capability of this model.

**Figure 6:**
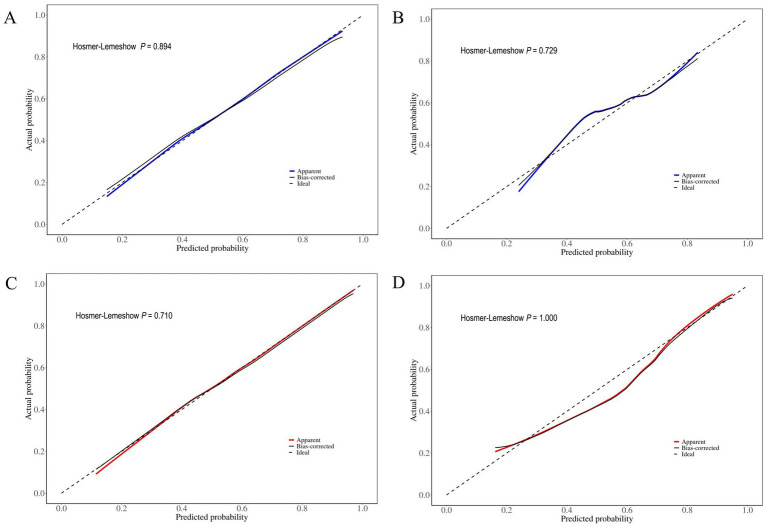
Calibration curves for **(A)** LS training cohort, **(B)** LS validation cohort, **(C)** LH training cohort, and **(D)** LH validation cohort. The dashed diagonal line (ideal) represents perfect prediction, the solid black line (bias-corrected) shows the adjusted calibration, and the apparent predictions are depicted by the blue solid line (LS model) and red solid line (LH model). The convergence of these curves demonstrates calibration performance: proximity between apparent (blue/red) and ideal lines reflects prediction accuracy, whereas agreement between apparent and bias-corrected (black) lines indicates model stability.

The calibration analysis demonstrated that our prediction models exhibited good overall predictive accuracy. As shown in the calibration plots (Figure 6), the predicted probabilities in the training cohort showed excellent agreement with observed probabilities, with the calibration curve closely following the ideal reference line (dashed diagonal). Although the validation cohort displayed a similar trend, its alignment with the ideal line was slightly less precise than that of the training cohort. Hosmer-Lemeshow test results confirmed satisfactory calibration performance for both models: the LS model showed no significant deviation between predicted and observed probabilities in either the training (*p* = 0.894) or validation cohorts (*p* = 0.729); similarly, the LH model demonstrated good calibration in both the training (*p* = 0.710) and validation cohorts (*p* = 1.000). Notably, while the bias-corrected line (black solid line) and the apparent line (LS model: blue solid line; LH model: red solid line) showed high concordance in the training cohort, minor deviations were observed in the validation cohort. These findings suggest a slight decrease in predictive performance during external validation, though the overall calibration remained within acceptable limits for clinical application.

**Figure 7:**
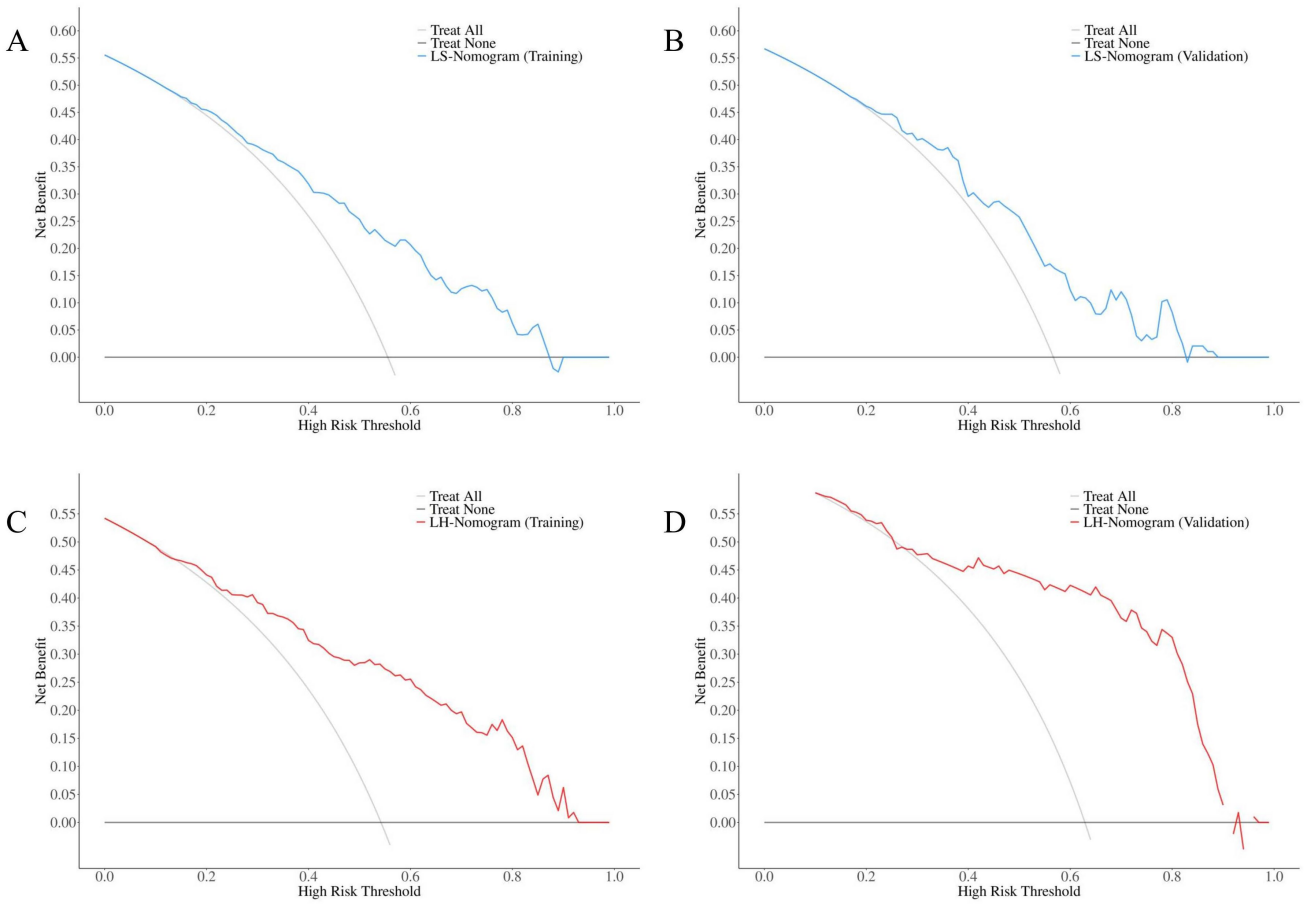
Decision curve analysis for **(A)** LS training, **(B)** LS validation, **(C)** LH training, and **(D)** LH validation cohorts, showing the nomogram models’ net benefit (blue/red solid lines) versus reference strategies: “Treat All” (diagonal solid line, indicating treat-all approach with inherent over-treatment) and “Treat None” (horizontal solid line, representing no-intervention strategy). The models’ curves exceed both reference lines across most threshold probability ranges, demonstrating significant net benefit advantages in clinically relevant probability intervals.

The decision curve analysis demonstrated the clinical utility of our prediction models. For the LS model, both the training cohort (threshold probability range: 0–0.85) and validation cohort (range: 0–0.82) showed positive net clinical benefit. Similarly, the LH model exhibited excellent clinical applicability, providing net benefit across threshold probability ranges of 0.15–0.92 (training cohort) and 0.27–0.91 (validation cohort) (Figure 7). These findings indicate that both models offer valuable clinical decision-making guidance across wide threshold probability ranges.

**Figure 8:**
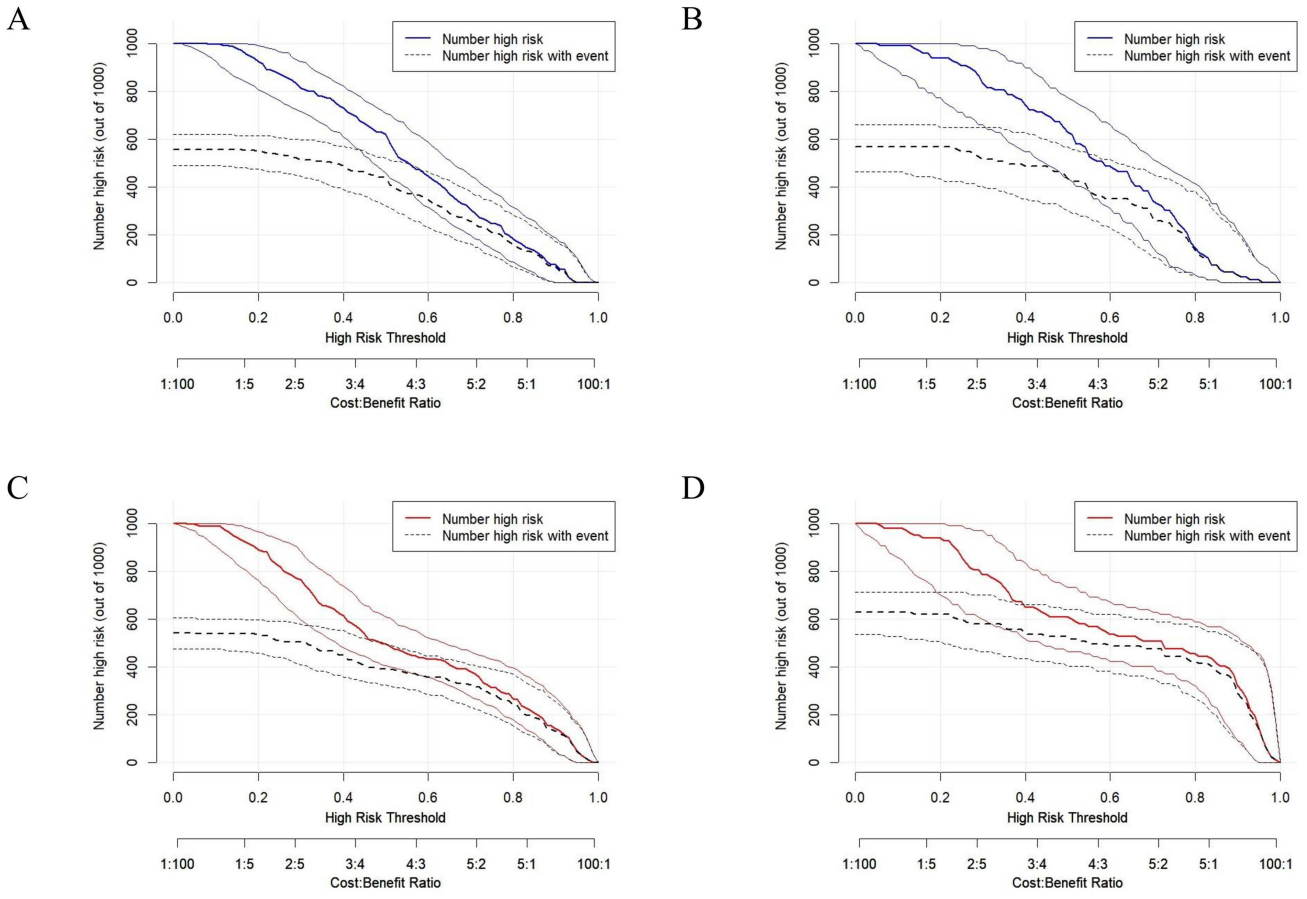
Clinical impact curves for **(A)** lumbar spine training cohort, **(B)** lumbar spine validation cohort, **(C)** left hip training cohort, and **(D)** left hip validation cohort. Axes: x = High Risk Threshold; y = *Number high risk (out of 1,000)* (estimated individuals classified as high-risk per 1,000 patients). Curves: predicted events by lumbar spine (blue) and left hip (red) nomograms; observed events (black).

The clinical impact curve analysis provided a visual assessment of the model’s practical clinical utility, demonstrating high concordance between the number of high-risk individuals identified by the model and the actual occurrence of low BMD at risk thresholds >60% (Figure 8). Curve morphology analysis revealed that the predicted curve (LS model: blue; LH model: red) and the actual observed curve (black) maintained essentially parallel trajectories in the >60% threshold range, indicating stable predictive accuracy of the model, while the minimal vertical separation between the two curves reflected the model’s relatively small margin of error. This consistent performance across higher risk thresholds suggests robust clinical applicability for identifying patients who would most benefit from targeted interventions.

## 4 Discussion

By utilizing readily available clinical data, the present study developed a nomogram prediction model to accurately assess the likelihood of low BMD at various sites (LS and LH) in male individuals with AS. Following a thorough and methodical evaluation, both prediction models were found to exhibit strong discriminative ability, high accuracy, and substantial clinical benefit. These models have the potential to support clinicians in more effectively and efficiently screening for low BMD in male AS patients.

The research findings identified age at onset, BMI, SUA levels, and hip involvement as common predictors for low BMD in both the LS and the LH. These factors have been consistently reported in previous literature as being strongly linked to low BMD in AS patients. The results indicated that a younger age at onset was linked to an elevated risk of low BMD in individuals with AS. In juvenile-onset AS (JoAS), chronic inflammation disrupts normal bone metabolism before the completion of skeletal development. This persistent inflammatory state stimulates osteoclast activity while suppressing osteoblast function, ultimately impairing bone mass accumulation and heightening the risk of low BMD (14). Furthermore, an earlier disease onset signifies a prolonged duration of skeletal involvement. As the disease advances, issues related to low BMD may become more pronounced, potentially elevating the risk of osteoporosis (OP) and fractures in the later stages. Several studies have affirmed that low BMD can manifest even in the early stages of AS (3, 15), underscoring the importance of timely BMD monitoring and consideration of appropriate interventions for JoAS patients to mitigate progressive bone loss.

The study identified BMI as an independent protective factor against low BMD in male AS patients, which aligns with previous reports of a positive BMI-BMD association (16–18). The osteoprotective mechanisms of BMI may involve mechanical stress stimulation, hormonal regulation, and nutritional status. In accordance with Wolff’s law, increased body weight enhances mechanical loading on bones, thereby promoting osteogenesis (19). Additionally, adipose tissue-derived factors such as estrogen and adiponectin participate in bone metabolism regulation (20). Moreover, higher BMI reflects better nutritional status, with adequate intake of protein, calcium, and vitamin D playing crucial roles in maintaining bone density. To further investigate potential heterogeneity in BMI’s effects, our lipid-stratified analyses revealed significant protective associations in both low-TC (OR = 0.86, *p* = 0.047) and high-TC subgroups (OR = 0.77, *p* = 0.019), with consistent LH protection observed in low/moderate-TC subgroups (OR = 0.77/0.74, both *p* < 0.01). These findings suggest that BMI’s protective effects may operate independently of lipid metabolism status (interaction *p* > 0.4). However, it must be emphasized that BMI, as a composite measure of weight and height, has inherent limitations – it cannot differentiate the heterogeneous contributions of lean mass versus fat mass (21), nor does it account for variations in fat distribution (e.g., visceral fat accumulation) (22) or regulatory effects of related hormones (e.g., testosterone, PTH) (23). Therefore, while our study supports the overall protective role of BMI, future research should incorporate DXA-based body composition analysis, waist-to-height ratio measurements, and metabolic marker assessments to more precisely evaluate the BMI-BMD relationship and inform individualized clinical decision-making.

Our study identified SUA as a protective factor against low BMD in AS patients, which is consistent with previous research. A cross-sectional study demonstrated a positive correlation between SUA levels and LS BMD in young male AS patients (24), while a Chinese multicenter study further confirmed SUA’s protective effects against osteopenia and osteoporosis (25). The bone-protective mechanisms of SUA may involve its anti-inflammatory and antioxidant properties. Current evidence suggests oxidative stress as a potential mechanism underlying osteoporosis (26). As a potent endogenous antioxidant, SUA may inhibit osteoclast differentiation and promote osteoblast activity by scavenging oxygen free radicals (27). This was validated in an *in vitro* study showing that SUA dose-dependently reduced osteoclast formation and decreased ROS production in osteoclast precursors (28). Additionally, Lai et al. found that physiological concentrations of SUA exerted anti-inflammatory effects by suppressing pro-inflammatory cytokine expression and cartilage-degrading enzyme production, thereby preventing cartilage damage and bone erosion (29). However, some studies have reported that intracellular urate in hyperuricemia may stimulate superoxide and free radical formation, leading to oxidative damage and inflammatory stress that disrupts bone remodeling (30, 31). This “double-edged sword” effect suggests a potential U-shaped relationship between SUA and BMD. As our study population had SUA levels primarily within the physiological range, we were unable to fully explore this U-shaped association. This limitation highlights the need for future large-scale prospective studies focusing specifically on different SUA level intervals (particularly the >420 μmol/L subgroup) to comprehensively elucidate the dose–response relationship between SUA and bone metabolism.

To clarify whether the protective effect of SUA is influenced by disease progression, we conducted further subgroup analyses. The results demonstrated that SUA’s protective effects remained stable in both early-stage AS (LS: OR = 0.99, *p* = 0.022; LH: OR = 0.95, *p* = 0.023) and advanced-stage AS (LS: OR = 0.99, *p* = 0.007; LH: OR = 0.99, *p* = 0.004). Although the effect sizes were modest, interaction analysis revealed no significant modification by disease stage (*p* > 0.8). These findings suggest that SUA’s effect may not be stage-dependent, showing relatively consistent impacts on BMD across different phases of the disease.

The results suggested that hip involvement served as an IRF for low BMD in AS patients, which aligns with prior findings (32). A number of studies have demonstrated that hip involvement correlates with more extensive spinal radiographic damage, elevated disease activity, prolonged course of disease, and diminished physical function (33–35). Spinal radiographic damage is closely linked to disease progression, particularly in later stages when the formation of bone bridges and spinal fusion occurs. These processes reduce mechanical stress stimulation on the bones, thereby exacerbating bone loss (36). Disease activity in AS patients is strongly associated with systemic inflammation. In states of heightened inflammation, immune cells secrete a range of cytokines, encompassing TNF, IL-6, IL-1, and IL-17, which activate the OPG/RANKL/RANK signaling pathway, influencing osteoclasts. This activation leads to increased bone resorption and a concomitant decrease in bone formation (37, 38). AS, as a chronic inflammatory condition, implies that prolonged course of disease not only reflects long-term inflammatory disruption of bone metabolism but also entails additional factors, such as aging and declining physical function, which negatively affect BMD.

In addition to the four common predictors previously discussed, serum calcium was identified as an IRF for low BMD in the LS in this study. Serum calcium exists in the blood in both free and bound forms, serving a function in bone mineral deposition and serving as a marker of bone metabolism. During bone metabolism, alterations in blood calcium levels regulate the secretion of parathyroid hormone (PTH) and calcitonin through feedback mechanisms, indirectly influencing osteoblast and osteoclast activity. This process facilitates a dynamic equilibrium exchange between bone calcium and blood calcium, thus contributing to bone mineral deposition. An animal experiment demonstrated that compared to the negative control cohort, the AS, OP, and AS + OP cohorts exhibited markedly higher levels of serum calcium and tartrate-resistant acid phosphatase (*p* < 0.05) (39). It is hypothesized that the observed elevation in serum calcium levels results from bone metabolic imbalance in AS, where osteoclast-mediated bone resorption surpasses bone formation. However, the precise mechanism underlying this process warrants further investigation and validation.

In conclusion, nomograms were developed to predict low BMD at different sites (LS and LH) in male AS patients. When compared to prior nomogram models (32), the current models are more specific and practical. These models are designed with a focus on the male AS population, effectively eliminating the confounding influences of gender and menopause during model construction. Furthermore, following their development and evaluation, the models have been made publicly accessible online. In clinical settings, healthcare professionals can easily utilize these models via the internet to assess the risk of low BMD, thereby enabling early prevention and personalized treatment for male AS patients with low BMD.

However, this study has several limitations. First, the single-center retrospective design and modest sample size (*n* = 322) may restrict the statistical power of interaction analyses and potentially introduce selection bias—for example, underestimating the prevalence of hyperuricemia. Although chronological age stratification was included, potential recall bias in self-reported symptom onset and substantial missing data (40%) for the exact diagnostic age could compromise the precision of course of disease-related analyses. Second, crucial clinical variables such as malnutrition, fracture history, testosterone, parathyroid hormone (PTH), and the Ankylosing Spondylitis Disease Activity Score (ASDAS) were not incorporated, which may undermine the model’s comprehensiveness—especially for patients with metabolic abnormalities or advanced disease stages. Additionally, while an internal validation cohort was used to assess model performance, the absence of external cohort validation warrants prudence when generalizing these results to broader populations. Finally, interpreting the protective role of BMI is hindered by the lack of DXA-based body composition data (e.g., lean vs. fat mass distribution) and obesity-related metabolic markers (e.g., insulin resistance), which might exert differential effects on bone metabolism. Future multicenter prospective studies integrating advanced imaging techniques, metabolic profiling, and standardized diagnostic datasets are essential to address these research gaps.

## 5 Conclusion

Drawing from the BMD results and clinical data of male AS patients, this study identified several factors as predictive of low BMD at the LS, including age at onset, BMI, serum calcium, SUA, and hip involvement (*p* < 0.05). Similarly, predictive factors for low BMD in the LH were found to include age at onset, BMI, SUA, and hip involvement (*p* < 0.05). Based on these observations, nomogram prediction models were developed for both the LS^2^ and LH.^3^ These models aim to aid rheumatologists in conducting rapid screening screening of male patients with AS by utilizing simple and commonly available clinical indicators, thereby facilitating early prevention and personalized treatment strategies for low BMD and contributing to clinical translation.

## Data Availability

The raw data supporting the conclusions of this article will be made available by the authors, without undue reservation.

## Footnotes

^1^www.zstats.net

^2^Accessible at: https://asresearch.shinyapps.io/shiny1/.

^3^Accessible at: https://asresearch.shinyapps.io/shiny/.
