# Supplementary material for: Development and validation of a nomogram for predicting low bone mineral density in male patients with ankylosing spondylitis
Source: Front Med (Lausanne). 2025 May 9;12:1549653. doi: 10.3389/fmed.2025.1549653 (PMC12098366; doi:10.3389/fmed.2025.1549653)
Supplement: Supplementary file 1 [file Data_Sheet_1.doc]

Supplementary Material

# Development and Validation of a Nomogram for Predicting Low Bone Mineral Density in Male Patients with Ankylosing Spondylitis

**Xiaotong Yang1, Qin Cheng2, Yifan Li1, Hao Tang1,3, Xin Chen1, Lijun Ma1, Jing Gao1 and Wei Ji4***

*** Correspondence:** Wei Ji:[weiweiji1103@163.com](mailto:weiweiji1103@163.com)

# Supplementary Tables

Table S1. Multicollinearity diagnostics for lumbar spine low BMD prediction model.

| Variables | GVIF | Df | GVIF^(1/(2*Df)) |
| --- | --- | --- | --- |
| Chronological age groups | 2.291 | 2 | 1.23 |
| Hip involvement | 1.141 | 1 | 1.068 |
| Age at onset | 2.233 | 1 | 1.494 |
| BMI | 1.085 | 1 | 1.042 |
| Serum calcium | 1.235 | 1 | 1.111 |
| Serum uric acid | 1.164 | 1 | 1.079 |

# Supplementary Tables

Table S2. Multicollinearity diagnostics for left hip low BMD prediction model.

| Variables | GVIF | Df | GVIF^(1/(2*Df)) |
| --- | --- | --- | --- |
| Sacroiliitis average | 1.417 | 4 | 1.045 |
| Hip involvement | 1.361 | 1 | 1.166 |
| Age at onset | 1.084 | 1 | 1.041 |
| Course of disease | 1.298 | 1 | 1.139 |
| BMI | 1.099 | 1 | 1.048 |
| Serum uric acid | 1.12 | 1 | 1.058 |

# Supplementary Tables

Table S3. Adjusted multivariate logistic regression for lumbar spine low BMD with confounder control.

| Variables | β | .S. | Z | P | OR (95%CI) |
| --- | --- | --- | --- | --- | --- |
| Intercept | 1.24 | 3.21 | 0.39 | 0.699 | 3.46 (0.01, 1861.61) |
| Age at onset | -0.05 | 0.02 | -2.2 | 0.028 | 0.96 (0.92, 0.99) |
| BMI | -0.14 | 0.06 | -2.49 | 0.013 | 0.87 (0.78, 0.97) |
| Serum calcium | 2.38 | 1.19 | 2 | 0.046 | 10.84 (1.05, 112.32) |
| Serum uric acid | -0.01 | 0 | -2.92 | 0.004 | 0.99 (0.99, 0.99) |
| Hip involvement |  |  |  |  |  |
| No |  |  |  |  | 1.00 (Reference) |
| Yes | 1.39 | 0.35 | 3.93 | <.001 | 4.01 (2.01, 8.03) |
| Chronological age groups |  |  |  |  |  |
| 18-44 |  |  |  |  | 1.00 (Reference) |
| 45-64 | -0.42 | 0.45 | -0.93 | 0.352 | 0.66 (0.27, 1.59) |
| ≥65 | 0.03 | 0.87 | 0.04 | 0.968 | 1.04 (0.19, 5.74) |
| TC groups |  |  |  |  |  |
| <3.70 |  |  |  |  | 1.00 (Reference) |
| 3.70-4.52 | -0.07 | 0.4 | -0.18 | 0.855 | 0.93 (0.42, 2.05) |
| >4.52 | -0.39 | 0.45 | -0.87 | 0.382 | 0.68 (0.28, 1.63) |
| TG groups |  |  |  |  |  |
| <0.89 |  |  |  |  | 1.00 (Reference) |
| 0.89-1.40 | 0.65 | 0.44 | 1.48 | 0.138 | 1.91 (0.81, 4.48) |
| >1.40 | 1.21 | 0.5 | 2.43 | 0.015 | 3.34 (1.26, 8.84) |
| Smoking history |  |  |  |  |  |
| Never |  |  |  |  | 1.00 (Reference) |
| Ever | -0.46 | 0.34 | -1.33 | 0.183 | 0.63 (0.32, 1.24) |
| Alcohol history |  |  |  |  |  |
| Never |  |  |  |  | 1.00 (Reference) |
| Ever | -0.06 | 0.37 | -0.17 | 0.863 | 0.94 (0.45, 1.94) |
| Patients on GC |  |  |  |  |  |
| No |  |  |  |  | 1.00 (Reference) |
| Yes | -1.17 | 0.85 | -1.37 | 0.17 | 0.31 (0.06, 1.65) |

β is the regression coefficient. TC, total cholesterol; TG, triglycerides.

# Supplementary Tables

Table S4. Adjusted multivariate logistic regression for left hip low BMD with confounder control.

| Variables | β | .S. | Z | P | OR (95%CI) |
| --- | --- | --- | --- | --- | --- |
| Intercept | 7.49 | 1.84 | 4.07 | <.001 | 1796.02 (48.82, 66070.24) |
| Age at onset | -0.03 | 0.02 | -2.13 | 0.033 | 0.97 (0.94, 0.99) |
| Course of disease | 0.02 | 0.03 | 0.6 | 0.546 | 1.02 (0.97, 1.07) |
| BMI | -0.23 | 0.06 | -3.65 | <.001 | 0.80 (0.71, 0.90) |
| Serum uric acid | -0.01 | 0 | -2.73 | 0.006 | 0.99 (0.99, 0.99) |
| Hip involvement |  |  |  |  |  |
| No |  |  |  |  | 1.00 (Reference) |
| Yes | 2.04 | 0.4 | 5.08 | <.001 | 7.72 (3.51, 16.98) |
| Sacroiliitis average |  |  |  |  |  |
| 2 |  |  |  |  | 1.00 (Reference) |
| 2.5 | -0.43 | 1.27 | -0.34 | 0.736 | 0.65 (0.05, 7.87) |
| 3 | 0.29 | 0.45 | 0.66 | 0.508 | 1.34 (0.56, 3.21) |
| 3.5 | 0.22 | 0.86 | 0.25 | 0.802 | 1.24 (0.23, 6.73) |
| 4 | 0.19 | 0.45 | 0.43 | 0.668 | 1.21 (0.50, 2.91) |
| TC groups |  |  |  |  |  |
| <3.70 |  |  |  |  | 1.00 (Reference) |
| 3.70-4.52 | 0.2 | 0.43 | 0.47 | 0.64 | 1.23 (0.52, 2.87) |
| >4.52 | 0.43 | 0.45 | 0.95 | 0.344 | 1.53 (0.63, 3.72) |
| TG groups |  |  |  |  |  |
| <0.89 |  |  |  |  | 1.00 (Reference) |
| 0.89-1.40 | 0.42 | 0.46 | 0.9 | 0.366 | 1.52 (0.61, 3.76) |
| >1.40 | 0.74 | 0.5 | 1.49 | 0.137 | 2.11 (0.79, 5.61) |
| Smoking history |  |  |  |  |  |
| Never |  |  |  |  | 1.00 (Reference) |
| Ever | 0.03 | 0.36 | 0.09 | 0.928 | 1.03 (0.51, 2.09) |
| Alcohol history |  |  |  |  |  |
| Never |  |  |  |  | 1.00 (Reference) |
| Ever | -0.02 | 0.39 | -0.06 | 0.951 | 0.98 (0.45, 2.12) |
| Patients on GC |  |  |  |  |  |
| No |  |  |  |  | 1.00 (Reference) |
| Yes | -0.46 | 0.87 | -0.52 | 0.6 | 0.63 (0.11, 3.50) |

β is the regression coefficient. TC, total cholesterol; TG, triglycerides.

# Supplementary Tables

Table S5. Comparison of effect size stability for core predictors between primary and confounder-adjusted models.

| Variable | Primary model OR (95% CI) | Confounder-adjusted model OR (95% CI) | OR change Rate | Stability conclusion |
| --- | --- | --- | --- | --- |
| Lumbar Spine Low BMD model |  |  |  |  |
| Age at onset | 0.96 (0.93, 0.99) | 0.96 (0.92, 0.99) | 0.00% | Highly stable |
| BMI | 0.90 (0.81, 0.99) | 0.87 (0.78-0.97) | -3.30% | Stable |
| Serum uric acid | 0.99 (0.99-0.99) | 0.99 (0.99-0.99) | 0.00% | Highly stable |
| Serum calcium | 12.19 (1.44-103.25) | 10.84 (1.05-112.32) | -11.10% | Stable |
| Hip involvement | 3.22 (1.71-6.05) | 4.01 (2.01-8.03) | 24.50% | Consistent direction |
| Left Hip Low BMD model |  |  |  |  |
| Age at onset | 0.97 (0.95, 0.99) | 0.97 (0.94, 0.99) | 0.00% | Highly stable |
| BMI | 0.81 (0.72-0.91) | 0.80 (0.71-0.90) | -1.20% | Highly stable |
| Serum uric acid | 0.99 (0.99-0.99) | 0.99 (0.99-0.99) | 0.00% | Highly stable |
| Hip involvement | 8.03 (4.01, 16.09) | 7.72 (3.51, 16.98) | -3.90% | Stable |
